# The synergistic antitumor effect of cinobufagin and cisplatin in human osteosarcoma cell line *in vitro* and *in vivo*

**DOI:** 10.18632/oncotarget.19554

**Published:** 2017-07-25

**Authors:** Guo Dai, Ling Yu, Jian Yang, Kezhou Xia, Zhengpei Zhang, Gaiwei Liu, Tian Gao, Weichun Guo

**Affiliations:** ^1^ Department of Orthopedics, Renmin Hospital of Wuhan University, Wuhan 430060, P. R. China; ^2^ Department of Orthopedic Oncology, Key Laboratory of Carcinogenesis and Translational Research, Ministry of Education, Peking University Cancer Hospital & Institute, Beijing 100000, P. R. China

**Keywords:** cinobufagin, cisplatin, osteosarcoma, combined treatment, apoptosis

## Abstract

Cisplatin (CDDP) has been shown to be a promising anticancer drug that is effective against many types of cancer, which include osteosarcoma (OS). However, its therapeutic application is restricted by its toxicity in normal tissues and by side effects caused in patients. Reduction of the toxicity of CDDP is necessary to improve cancer treatment. In the present study, we attempted to clarify how cinobufagin, a traditional Chinese medicine, enhances CDDP-induced cytotoxicity in OS cells. OS 143B cells were treated with cinobufagin and CDDP alone or in combination. After low dose combined treatments with cinobufagin and CDDP, the effects of these therapeutics on cell proliferation, apoptosis, cell cycle, migration, invasion, and involvement in Notch pathway, as well as tumor growth and metastatic capability were determined. It was found that the combination of low doses of cinobufagin and CDDP markedly inhibited cell activity, motility, and induced apoptosis and cell cycle arrest in S phase, as well as suppressing tumor growth, metastasis and prolonging longer survival of nude mice in OS xenograft models compared with the actions of either drug alone or vehicle. The results also demonstrated that cinobufagin plus CDDP significantly suppressed the Notch pathway. The anticancer mechanism of these two drugs may involve intervention in the Notch signaling, which may contribute to inhibit tumor growth. All of these results suggest that application of lower concentration cinobufagin plus CDDP could produce a synergistic antitumor effect and this finding warrants further investigation for its potential clinical applications in human OS patients.

## INTRODUCTION

OS is the most common malignant primary solid tumor of bone, particularly in children and young adults [1]. Standard treatment of OS involves neoadjuvant chemotherapy before definitive surgical resection (mostly limb-sparing or rarely amputation) of the primary tumor, followed by treatment with multiple chemotherapeutic agents or radiotherapy after operation [2, 3]. Nevertheless, a number of OS patients present with metastasis at diagnosis, and OS develops resistance to traditional chemotherapies, leading to treatment failure [4]. Thus, to further improve patients’ treatment, the development of novel, more effective and well tolerated therapeutic approaches against OS in clinical is urgent and important [5].

**Figure 1:**
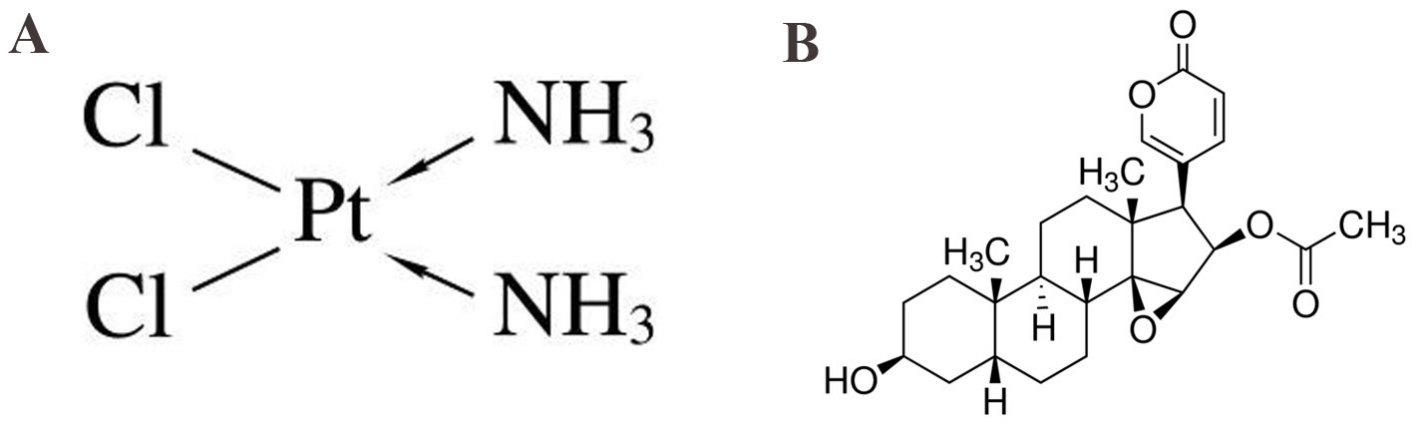
**(A)** Chemical structure of cisplatin (CDDP). **(B)** Chemical structure of cinobufagin.

Chemotherapy is the most common therapeutic strategy for tumor treatment. As early as four decades ago, cisplatin (CDDP) (Figure 1A) was discovered to cause tumor regression and elimination [6]. Generally, the mechanism by which CDDP kills tumors involved with inhibiting DNA replication by cross-linking with the purine bases on the DNA, leading to DNA damage, and finally inducing apoptosis in cancer cells [7, 8]. As one of the effective neoadjuvant chemotherapy drugs for OS treatment, CDDP has been confirmed to have a broad spectrum of cytotoxic and treat possible microscopic metastases, regress or eliminate tumors, and thus increase the opportunities and possibilities of limb-sparing surgery [9]. Interestingly, different OS patients have different sensitivities to neoadjuvant chemotherapy. Although it remains the pivotal chemotherapeutic agent to overcome inherent tumor resistance for OS therapy, acquired resistance to CDDP and considerable side effects are common and represent a major obstacle to effective treatment of OS. Thus, it is urgent to develop less toxic and more effective approaches to overcome these limitations or make up for its flaws [8].

One method for overcoming this toxicity and resistance is to use lower concerntrations of CDDP in combination with other complementary agents [10-14]. Combined therapy with traditional Chinese medicine is recommended and is regularly used in the clinic [11, 15-19] and many researchers have gradually found that traditional Chinese medicine combined with western medical treatment of tumors tend to achieve better results and to be accompanied with fewer side effects [20]. Pu *et al.* [11] found that Oldenlandia diffusa, a traditional Chinese medicine, combined with CDDP could inhibit proliferation and induce apoptosis in the human OS MG-63 cells, which might be mediated by Caspase activation. Lou *et al.* [21] demonstrated that Yu Ping Feng San, an ancient Chinese herbal decoction, can notably improve the cancer-suppressing effect of CDDP, which may be a consequence of the elevation of intracellular CDDP via drug transporters as well as the down-regulation of p62/TRAF6 signaling. Huang *et al.* [16] were the first to show that cinobufagin (Figure 1B) enhanced the CDDP induced killing effects on OS-732 cells, which might be related to up-regulation of Fas expression. Yang *et al.* [10] reported that the combination of low concentrations of sorafenib and CDDP has a synergistic antitumor effect when administered to Saos-2 cells, which reduces CDDP toxicity. Therefore, combination therapies of CDDP together with traditional Chinese medicine have been considered to overcome drug-resistance and reduce toxicity.

In this study, in addition to comparing the effects of cinobufagin and CDDP alone, we hypothesized that these two drugs may produce synthetic effect and thus be more effective than either agent administered alone. Therefore, in this work, we investigated whether combined low dose CDDP with cinobufagin may potentiate the growth inhibition of a human OS cell line *in vitro* and *in vivo* and its potential molecular mechanisms. Our data indicate that cinobufagin combined with CDDP is an effective treatment approach for human OS.

## RESULTS

### Anti-proliferative activity of cinobufagin and CDDP in 143B cells

**Figure 2:**
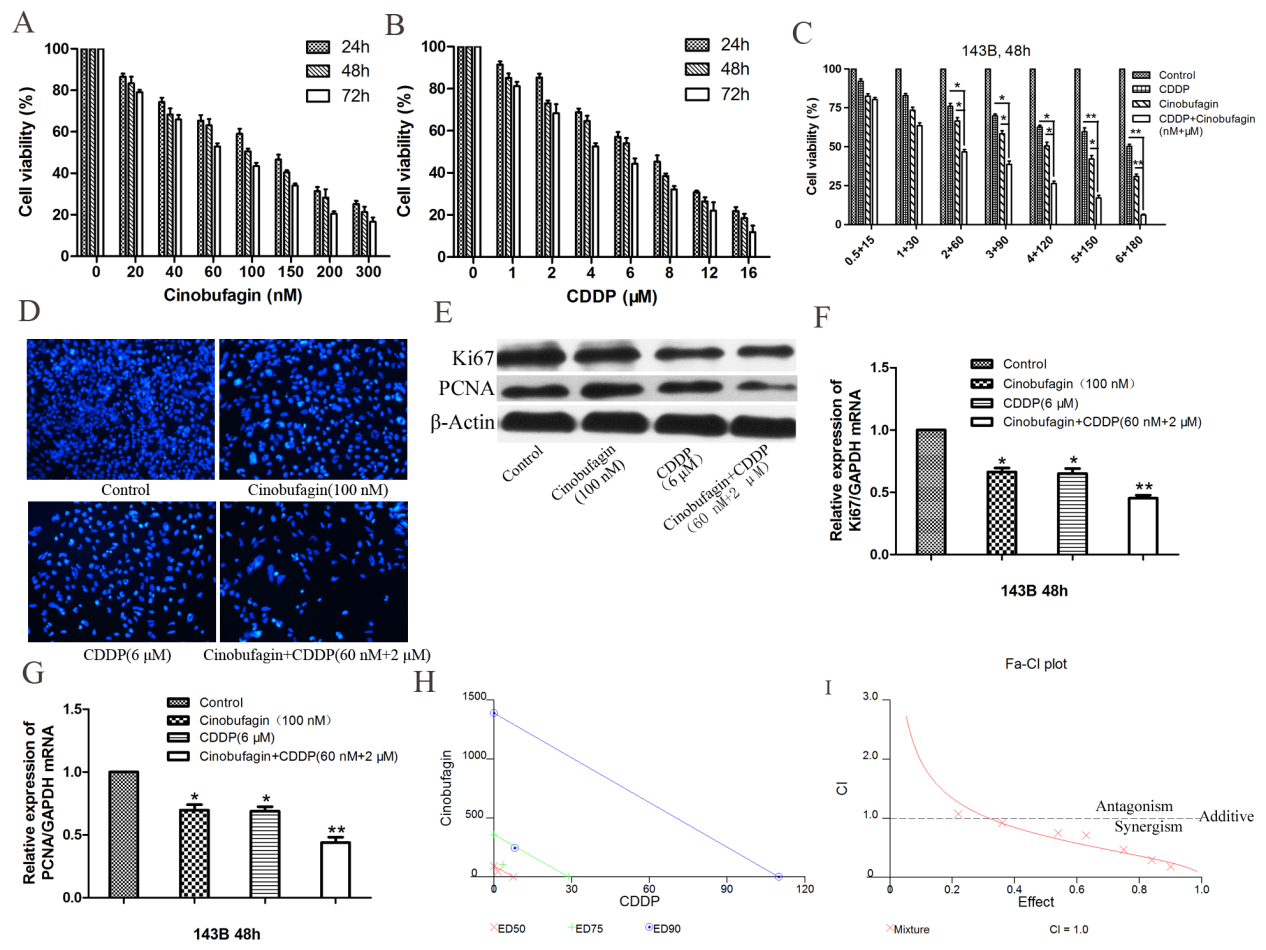
Cinobufagin synergistically enhanced cytotoxicity of CDDP in 143B cells *in vitro* **(A)** *In vitro* 143B cells were treated with cinobufagin at different concentrations (0 - 300 nmol/L) for 24, 48 and 72 h, and the cell viability was assessed by CCK-8 assay. **(B)** Cells were treated with CDDP at concentrations ranging from 0 to 16 μmol/L for 24, 48 and 72 h. **(C)** Either CDDP (0.5 – 6 μmol/L) or cinobufagin (15 - 180 nmol/L) alone or in combination at 1:30 (CDDP : Cinobufagin) fixed molar ratio treatment for 48 h. Cell proliferation was determined by CCK-8 assay. **(D)** 143B cells were treated with either cinobufagin or CDDP alone or in combination for 48 h and then been stained with DAPI, which showed that the combination group was significantly inhibited proliferation compared with the control group or using either agent alone (magnification,×200). **(E, F** and **G)** The mRNA and protein expression of Ki67 and PCNA in 143B cells which treated with cinobufagin and CDDP alone or in combination were analysis after 48 h. **(H)** Isobologram analysis of cytotoxicity of combined cinobufagin and CDDP in 143B cells. The diagonal line represents the isoeffect line of additivity. Points above this line indicate antagonism between drugs, and points below this line indicate synergy. **(I)** Combination index (CI) analysis of 143B cells treated with the combination of cinobufagin and CDDP. A combination index of 1.0 (dashed line) reflects additive effects, whereas values greater than and less than 1.0 indicate antagonism and synergy, respectively. Data are shown by means ± SD from at least three independent experiments. *P<0.05 vs. control, **P<0.01 vs. control.

The anti-proliferative effects of cinobufagin and CDDP alone in 143B cells were investigated using the CCK-8 assay. Cinobufagin and CDDP treatment resulted in a concertration- and time-dependent decrease in cell viability. Here, we demonstrated the survival rates of cinobufagin (0–300 nM) in 143B cells after 24, 48 and 72 h. Cinobufagin (100 nM) inhibited ∼ 50% proliferation of 143 cells (Figure 2A) and the half-maximal inhibitory concentration (IC_50_) values were ∼ 98–103 nM after 48 h (Table 1) treatment.

**Table 1:** IC_50_ values of cinobufagin and CDDP alone and in-combination in OS cell line

Cell line	IC_50_						
	Cinobufagin (nM)			CDDP (μM)			Cinobufagin+CDDP
	24h	48h	72h	24h	48h	72h	48h
143B	160 ± 3.24	100 ± 2.61	65 ± 1.72	8 ± 0.32	6 ± 0.25	5 ± 0.16	∼ 60 + 2

Cells were treated with varying concentrations of cinobufagin (0-300 nmol/L) and CDDP (0-16 μmol/L) alone for 24, 48 and 72 h and in combination at 1:30 (CDDP : Cinobufagin) molar ratio for 48 h. The growth inhibitory effect of cinobufagin and CDDP on OS cells were evaluated by CCK-8 assay. IC_50_ values shown here are the means ± SD from the data of three independent experiments.

We also verified anti-proliferative effect of CDDP (0–16μM) in 143B cells after 24, 48 and 72 h. IC_50_ values of CDDP was in range of ∼ 6 – 7 μM after 48 h (Figure 2B and Table 1) treatment. Our results showed that both cinobufagin and CDDP exert anti-proliferative activity in 143B cells with dose- and time-dependent characteristics.

### Cinobufagin synergistically enhanced the anti-proliferative activity of CDDP on 143B cells *in vitro*

The treatment of OS with CDDP is linked with its concentration-limiting toxicity [22] whereas cinobufagin is reported to be nontoxic up to 10 mg/kg body weight of nude mice [23]. Therefore, we aimed to improve the cytotoxic efficacy of CDDP by reducing its concentration in presence of cinobufagin. Based on the IC_50_ values of CDDP and cinobufagin, we performed dose-range experiments and selected 1 : 30 (CDDP : Cinobufagin) ratio for combination in 143B cells. We observed that CDDP (0.5–6.0 μM) alone inhibited ∼ 7–49% 143B cells proliferation after 48 h and cinobufagin (15–180 nM) alone exhibited ∼ 17–69% growth-inhibition. Interestingly, combination of CDDP and cinobufagin, at this fixed ratio, augmented growth-inhibition (∼ 19–93%) in 143B cells (Figure 2C). When CDDP combined with cinobufagin, the IC_50_ of CDDP and cinobufagin in 143B cells were found to decrease significantly, in a concentration-dependent manner respectively. As shown in Figure 2C and Table 1 the IC_50_ of CDDP and cinobufagin were 2 μM and 60 nM respectively, it is obviously that the concentrations of combined application were lower than those of their separate application. Based on these results, IC_50_ values of drugs were be selected respectively for further treatments throughout this study.

In order to further explore the combination effects of cinobufagin and CDDP on the proliferation of 143B cells, we conducted the DAPI (4’, 6-diamidino-2-phenylindole) staining assay. The results as shown in Figure 2D, treatment of the 143B cells with cinobufagin and CDDP alone or in combination for 48 h, which showed that the combination group was significantly inhibited proliferation compared with the control group and either agent alone.

To further confirm these results, two proliferation markers Ki67 and PCNA were detected using immunoblotting assay. The Western blotting results showed that treatment of the 143B cells with or without cinobufagin and CDDP alone and in combination for 48 h respectively, markedly decreased the protein expression levels of Ki67 and PCNA in the combination group when compared with the control group and separate application (Figure 2E). Similar to Western blotting results, the mRNA expressions of Ki67 and PCNA were also observed to decrease following combined treatment with cinobufagin and CDDP (Figure 2F and 2G).

To evaluate the enhanced efficacy obtained by combining cinobufagin and CDDP indicates synergism, isobologram analysis was performed, as shown in Figure 2H, most of the data points are positioned below the line of additive effects. Furthermore, the fraction-affected versus combination index (CI) curve shown in Figure 2I also exhibits a synergistic cytotoxic effect (CI < 1) of cinobufagin combined with CDDP, with CI values ranging from 0.1 to 0.9 at fixed molar ratio drug combination from IC_30_ to IC_90_ indicating the synergistic effect of cinobufagin and CDDP on 143B cells (Table 2, Figure 2I). These analysis reflects the results observed in Figure 2C. Cinobufagin plus CDDP resulted in an appreciable dose reduction index (DRI), which ranged from a 2.9- to 7.2-fold dose reduction for both agents (Table 3). Altogether these results reflected that when CDDP plus cinobufagin could yield a much greater proliferation inhibition than either agent alone and also showed the efficacy of cinobufagin to improve CDDP-mediated cytotoxicity but reducing its concentration.

**Table 2:** Combination index (CI) values for CDDP and cinobufagin in combination

Cell line	CDDP. (μM)	Cinobufagin (nM)	Molar ratio (CDDP:cinobufagin)	CI
143B	0.5	15	1:30	1.080
	1	30		0.925
	2	60		0.749
	3	90		0.712
	4	120		0.473
	5	150		0.297
	6	180		0.184

CI signifies a quantitative measure of the degree of drug interaction, calculated by using CalcuSyn software. The CI > 1 indicates antagonism; CI = 1 indicates additive effects; The CI < 1 indicates synergy. CI values < 1 indicated that interaction between CDDP (1 – 6μmol/L) and cinobufagin (30 – 180 nmol/L) was synergistic in 143B cells.

**Table 3:** Dose reduction index (DRI) values for CDDP in combination with cinobufagin

Cell line	Drug:compound	Molar ratio (CDDP:cinobufagin)	DRI(75% fraction affected level)
143B	CDDP:Cinobufagin	1:30	7.207

The DRI signifies the fold decrease of CDDP as a result of synergistic combination in compared with the concentration of a single agent needed to achieve the same effect. Concentrations of CDDP were reduced∼7 folds in 143B to achieve a 75% inhibition of cell proliferation when the cells were exposed to combination treatment at fixed molar ratios after 48 h.

### Enhanced cell-cycle arrest by combination of cinobufagin and CDDP

**Figure 3:**
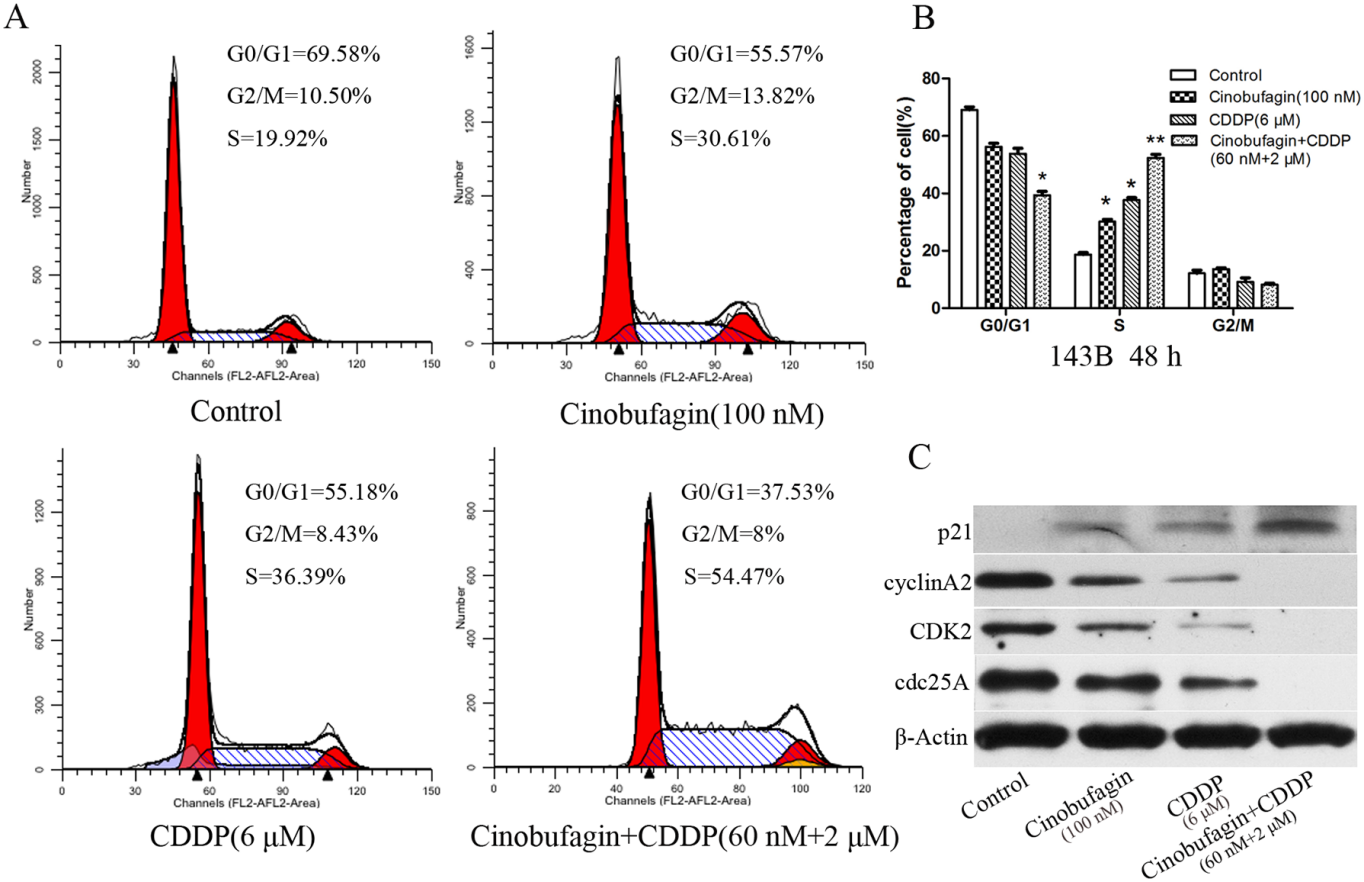
Effect of cinobufagin and CDDP alone or in combination on the cell cycle of 143B cells **(A)** Flow cytometry histograms of Cell DNA content distribution in each phase after treatment with cinobufagin and CDDP alone or in combination after 48 h showing S-phase arrest. **(B)** Percentage of cells distributed in each phase of the cell cycle. **(C)** After treatment as in A, proteins were extracted from cultured 143B cells and probed with appropriate dilutions of specific antibodies. Representative results of p21, Cyclin A2, CDK2, and cdc25A protein levels were as determined by a Western blot analysis. Data are shown by means ± SD from at least three independent experiments. *P<0.05 vs. control, **P<0.01 vs. control.

In order to investigate the effects of cinobufagin and CDDP on cell cycle, we made use of flow cytometry (FCM) analysis to detect the number of cells in each phase of cell cycle. 143B cells were incubated with cinobufagin and CDDP alone or in combination for 48 h and cell cycle distribution was analysis by FCM. The results indicated that the combinational group effectively arrested the cell cycle in S phase compared with the control group (P<0.01) (Figure 3A and 3B). Cinobufagin plus CDDP was leading to an even much greater percentage of arrest in S phase than the higher concerntration of either agent alone. To study the molecular mechanisms underlying cinobufagin- and CDDP-induced S-phase arrest, the expressions of cell cycle-related proteins in response to cinobufagin and CDDP treatment was determined by western blot analysis. As shown in Figure 3C, the result showed the level of p21 was significantly increasd in combinational cinobufagin- and CDDP-treated 143B cells. Cdc25A is a critical convergence point with the DNA-damage checkpoint, so to explore the complex network connecting cdc25A and CDK activity is essential. We examined protein levels of cdc25A, Cyclin A2 and CDK2, three known proteins that were found to decrease in single and combined application of cinobufagin- and CDDP-treated 143B cells, especially for combined group. These results demonstrated that cinobufagin and CDDP inhibited OS cell proliferation was related to S phase cell cycle arrest.

### Enhanced apoptosis by combination of cinobufagin and CDDP

**Figure 4:**
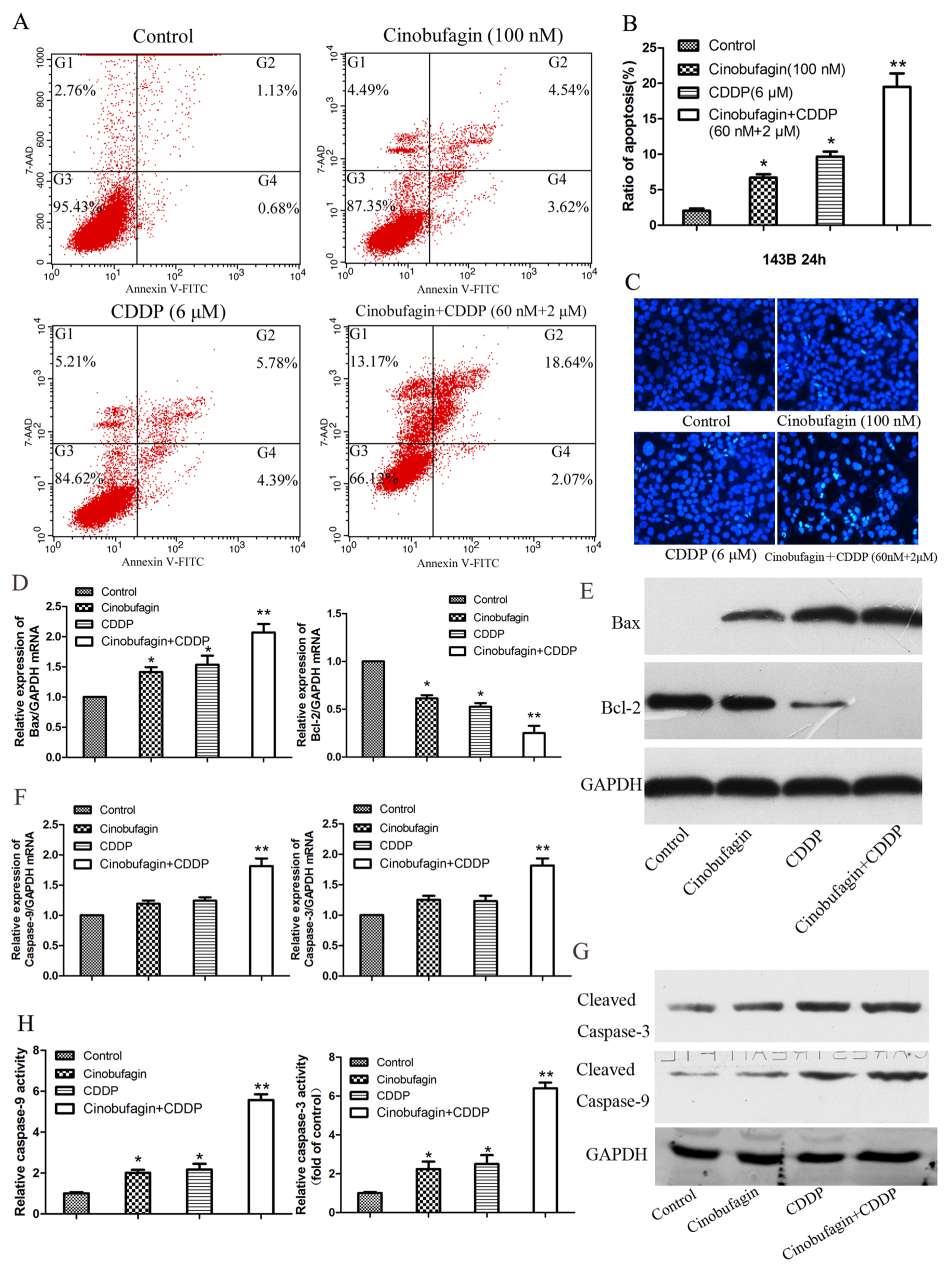
Effect of cinobufagin and CDDP alone or in combination on the cell apoptosis and caspase activation of 143B cells **(A)** Flow cytometry histograms of Cell apoptosis distribution after treatment with cinobufagin and CDDP alone or in combination after 24 h. **(B)** Quantitative analysis of apoptosis of 143B cells as showing in (A). **(C)** Hoechst 33258 staining of 143B cells treated with cinobufagin (100 nmol/L),CDDP (6 μmol/L), or the combination of both agents (60 nmol/L + 2 μmol/L) for 48 h. Apoptotic cells were identified by the presence of bright-blue fluorescent and highly condensed or fragmented nuclei (×200). **(D-G)** The mRNA and protein expression of Bax, Bcl-2, Caspase-3 and Caspase-9 in 143B cells which treated with cinobufagin and CDDP alone or in combination were analysis after 48 h. **(H)** Caspase-3 and caspase-9 activity were determined following treatment with cinobufagin and CDDP alone or in combination. Data are expressed as the mean ± SD of three independent experiments. *P<0.05 vs. control, **P<0.01 vs. control.

FCM revealed that the apoptotic rate in the combined application of cinobufagin (60 nM) and CDDP (2 μM) was markedly higher than that in the control group (P<0.01) and the monotherapy group, and the apoptotic rate of each monotherapy group was higher than the control group (P<0.05, Figure 4A and 4B). Additionally, the apoptosis elicited by cinobufagin and CDDP exposure was observed visually as well. When cells treated combined with cinobufagin and CDDP showed much more bright-blue fluorescent and condensed nuclei than untreated cells and single application of cinobufagin or CDDP (Figure 4C), which also indicated apoptosis induction as well. All the above results demonstrated that combined application of cinobufagin and CDDP could significantly induce apoptosis of 143B cells.

In order to investigate the underlying mechanism of pro-apoptosis effect of combination with cinobufagin and CDDP, the expressions of apoptosis-related mRNA and proteins were investigated. We tested the expressions of Bax, Bcl-2, Caspase-9, and Caspase-3 in 143B cells after the cells were exposed to with or without cinobufagin (100 nM) and CDDP (6 μM), and combination of cinobufagin (60 nM) and CDDP (2 μM) for 48 h respectively. The results showed that the levels of pro-apoptotic mRNA and protein (Bax) in combined group were higher than the monotherapy and control group. Conversely, the level of anti-apoptotic mRNA and protein (Bcl-2) in combination group were lowest (Figure 4D and 4E). The levels of Caspase-3 and Caspase-9 in single application of cinobufagin or CDDP were elevated, but the levels of Caspase-3 and Caspase-9 in the combination group was much higher than the control and monotherapy group (Figure 4F and 4G). Subsequently the anticancer effect of cinobufagin and CDDP treatment on Caspase-3 and Caspase-9 activity in OS cells were investigated. Compared with control group, treatment with cinobufagin (100 nM) or CDDP (6 μM) significantly induced Caspase-3 and Caspase-9 activity of 143B cells at 48 h (P < 0.05), furthermore activity of Caspase-3 and Caspase-9 increased most obviously in the combined group (P < 0.01, Figure 4H).

### Combination of cinobufagin and CDDP decreased more cell motility

**Figure 5:**
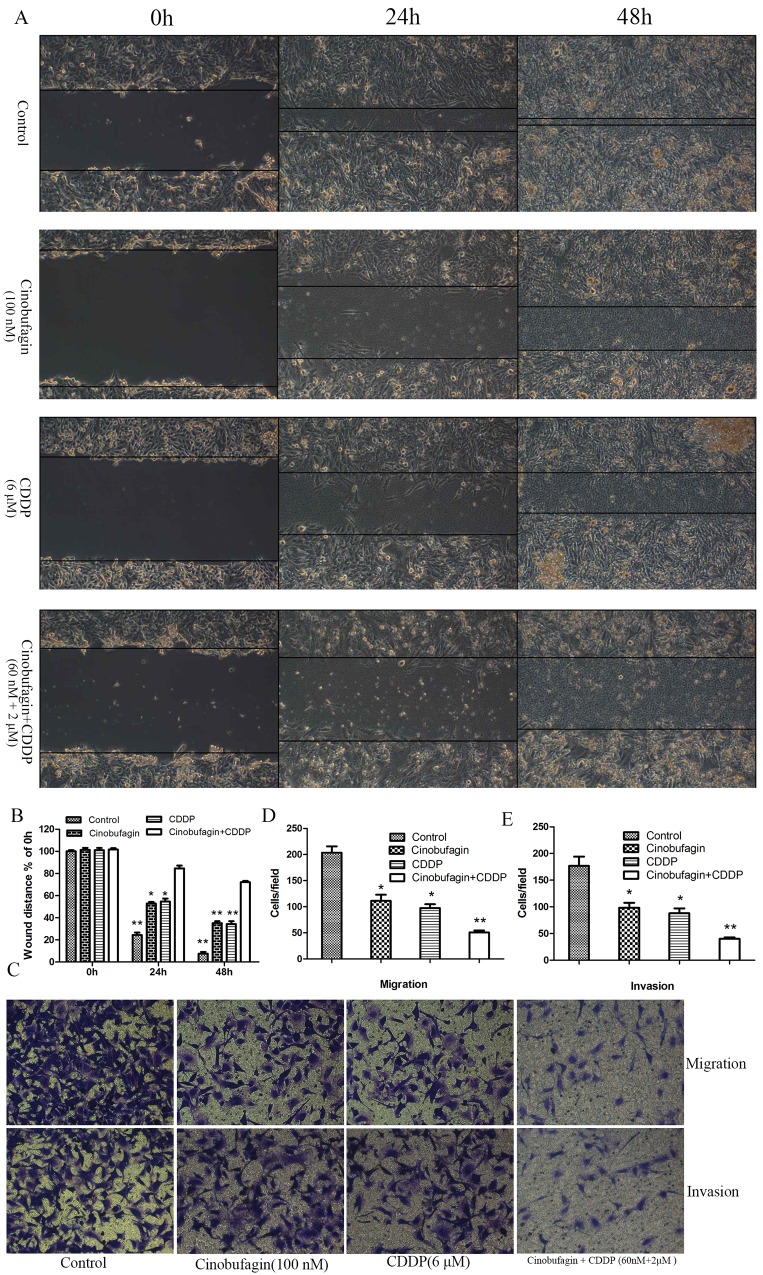
Effect of cinobufagin and CDDP alone or in combination on the migration and invasion of 143B cells **(A)** Cell migration was determined by wound-healing assay following treatment with cinobufagin and CDDP alone or in combination. **(B)** The wound distance of migration was calculated. **(C)** Representative images of the Transwell assay without (top panel) or with (bottom panel) Matrigel coating following treatment with cinobufagin and CDDP alone or in combination. **(D)** Migration assay results showing the number of 143B cells that had migrated through the non-Matrigel coated filters. **(E)** Invasion assay showing the number of cells that had passed through the Matrigel-precoated filters. The cell counts presented are the mean values/field from at least five randomly selected low-power fields (magnification, ×200) from three independent experiments, data are expressed as the mean ± SD. *P<0.05 vs. control, **P<0.01 vs. control.

We examined whether cinobufagin and CDDP plays a role in OS cell migration, invasion and metastasis. First, the migration potential of 143B cells were investigated by a wound healing assay. Cells in the combination or monotherapy group migrated markedly less than those in the control group (Figure 5A and 5B). And most importantly, cell migration in the combination group was lower than either single drug alone (Figure 5A and 5B). To further confirm this, we performed *in vitro* Transwell cell migration and invasion assays. There were significantly decreased numbers of migrating and invasive cells of combination group compared with the single application group and control group (Figure 5C - 5E). These results emphasized the inhibitive effect of combined effect on migration and invasion in OS cells *in vitro*.

**Figure 6:**
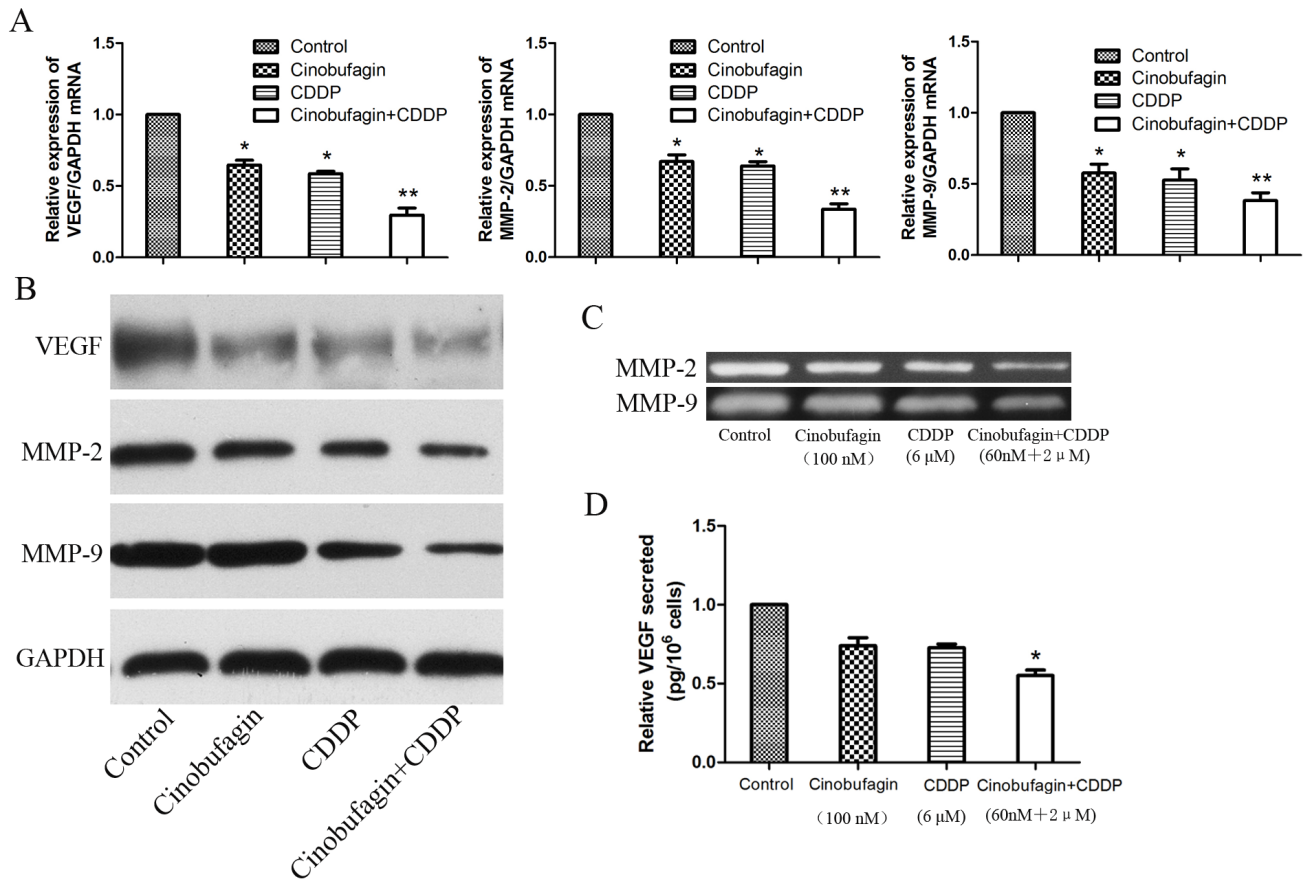
Cinobufagin and CDDP synergistically decrease the expression levels and activities of VEGF, MMP-2 and MMP-9 **(A-B)** RT-PCR and Western blot analysis of VEGF, MMP-2 and MMP-9 mRNA and proteins expression following treatment with cinobufagin and CDDP alone or in combination. GAPDH was used as an internal control. **(C)** Gelatin zymography analysis the activities of MMP-2 and MMP-9. **(D)** ELISA assay were performed to detect the VEGF secreted. Data were expressed as mean ± SD from at least three independent experiments.*P<0.05 vs. control, **P<0.01 vs. control.

To explore whether the inhibition of migration and invasion effect of cinobufagin and CDDP was associated mechanistically with vascular endothelial growth factor (VEGF) and matrix metalloproteinases (MMPs), the expression of VEGF, MMP-2 and MMP-9 were investigated in all group. Compared to control and single application group, the mRNA and proteins expression of VEGF and MMPs were significantly reduced in combination treated group (Figure 6A and 6B). Then, we investigated whether MMPs activities could be depressed by combination therapy. Gelatin zymography analysis showed that the activities of MMP-2 and MMP-9 were decreased in combination treatment group compared with control and single application group (Figure 6C). In addition, we also noted that combination therapy could decrease the levels of VEGF secreted in the culture medium, which assessed by Enzyme-Linked Immunosorbent Assay (ELISA) assay (Figure 6D). These data indicated that combined application of cinobufagin and CDDP could effectively restrain the expression and activities of VEGF, MMP-2 and MMP-9 in OS cells compared to the monotherapy groups and control group.

### Effects of cinobufagin and CDDP on Notch signaling pathway in 143B cells

**Figure 7:**
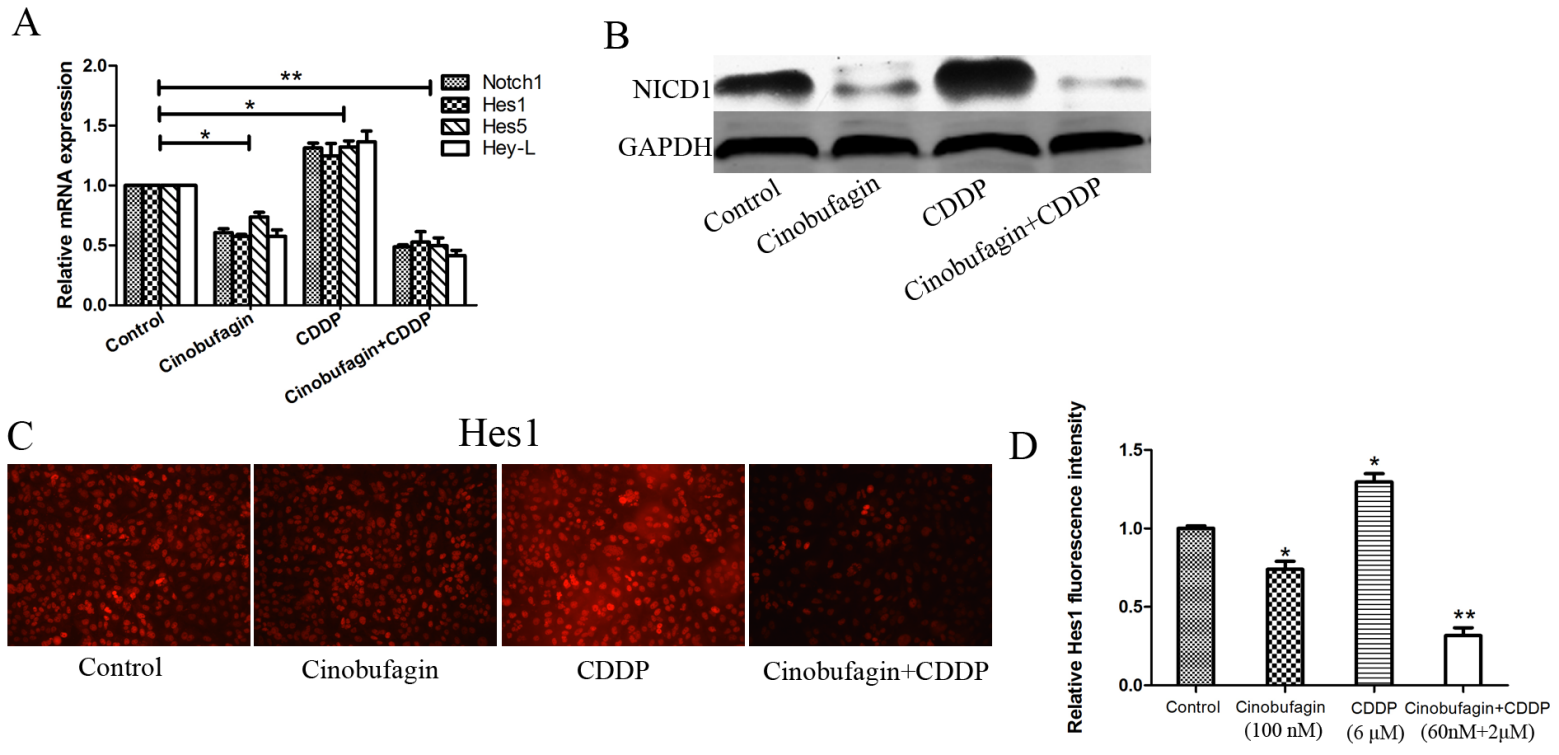
Effect of cinobufagin and CDPP alone or in combination on Notch signaling in 143B cells **(A-B)** RT-PCR and Western blot analysis the expression of Notch and its target genes following treatment with cinobufagin and CDDP alone or in combination. GAPDH was used as an internal control. **(C)** Immunofluorescence assay to detect the expression of Hes1 following treatment with cinobufagin and CDDP alone or in combination. **(D)** Relative quantification of Hes1 protein by fluorescence intensity analysis. Data are shown by means ± SD from three independent experiments. *P<0.05 vs. control, **P<0.01 vs. control.

In order to further understand the specific molecular mechanism involved in cinobufagin and CDDP synergistically inhibited proliferation and induced apoptosis and suppressed motility, the Notch signaling pathway was investigated. In our previous study, The effects of cinobufagin were known to down-regulation Notch signaling pathway [24] and CDDP alone lead to opposite effect [4]. Therefore, we evaluated the effects of cinobufagin and CDDP alone or in combination on the expression of Notch1 and its target genes Hes1, Hes5, Hey-L by using real-time RT-PCR after treatment 48 h with their respective IC_50_ values (Figure 7A). Compared with control, there were reduction of Notch1, Hes1, Hes5 and Hey-L mRNA levels after cinobufagin treatment, which suggested that cinobufagin could lead to transcriptional inactivation of Notch signaling pathway in OS cells. However, application of CDDP alone resulted in increased expression of Notch1 and its target genes. These results were similar to our previous studies. Interestingly, when combination of cinobufagin and CDDP, the expression of Notch1 and its target genes was not only decreased but also lower (Figure 7A). Consistent with the real-time RT-PCR data, western blotting showed the similar results from protein expression levels (Figure 7B). In order to further confirm these results, we conducted immunofluorescence assay to detect Hes1 protein expression. The results showed that application of cinobufagin alone led to decrease the fluorescence intensity of Hes1, but when using of CDDP alone lead to opposite result. And the combination group showed the lowest fluorescence intensity (Figure 7C and 7D).

Together, we concluded that cinobufagin downregulates Notch pathway activity due to CDDP treatment which should have been elevated. So, we have reason to speculate that cinobufagin can play a synergistic anticancer effect with CDDP by interfering with Notch signaling pathway in OS cells and thus inhibiting tumor cell proliferation and increasing chemosensitivity.

### Cinobufagin and CDDP inhibit tumor growth and metastasis and prolong survival time in xenograft mouse model

**Figure 8:**
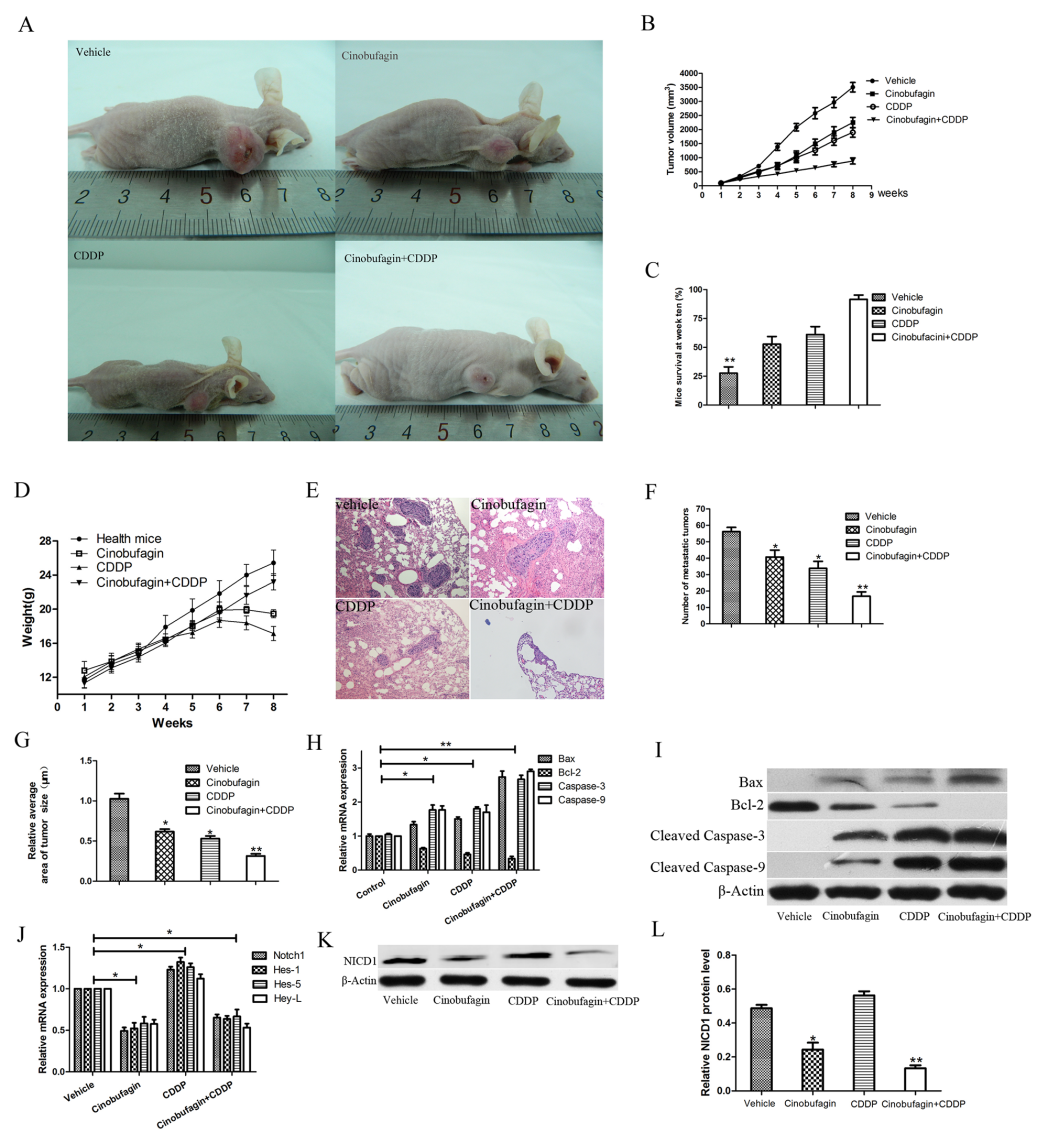
Antitumor activity of cinobufagin combined with CDPP in BALB/c-nu mice bearing 143B cells **(A)** Representative images of 143B xenograft treated and untreated with cinobufagin and CDDP alone or in combination were shown. **(B)** The tumor growth were recorded and compared (volume in mm^3^, recorded every week). **(C)** The survival rates of mice at week 10 were compared. **(D)** The body weight (in grams, recorded every week) were recorded and compared. **(E-G)** HE staining (magnification, ×100) indicated the lung metastases and its number and relative average size were calculated. **(H** and **I)** The mRNA and proteins expression of Bax, Bcl-2, Caspase-3 and Caspase-9 in xenograft tumor tissues which treated with cinobufagin and CDDP alone or in combination. **(J)** The Notch and its target genes expression were tested using RT-PCR. **(K)** Western blot analysis of NICD1. **(L)** Relative quantification of NICD1 protein by densitometric analysis. Data are shown by means ± SD from at least three independent experiments. *P<0.05 vs. vehicle, **P<0.01 vs. vehicle.

To further determine a role of cinobufagin and CDDP in progression of OS, the antitumor effects of cinobufagin and CDDP alone or in combination in human xenograft tumor models were evaluated. The male BALB/c-nu/nu nude mice were inoculated subcutaneous with 5×10^6^ 143B cells in 0.2 mL of serum-free medium at the right or left forelimb. When the tumors reached a size of about 50-100 mm^3^ (10-15 days after transplantation), mice were treated with vehicle, cinobufagin, CDDP and cinobufagin combined with CDDP respectively. Xenografted OS tumors received 5 mg/kg of cinobufagin. Cinobufagin was injected intraperitoneally (i.p.) every other day for 3 week [25, 26]. During which, the CDDP (4 mg/kg) was administered i.p. twice a week for 3 weeks. And the combination therapy group received two aforementioned treatment methods, but with lower doses with cinobufagin (3 mg/kg) and CDDP (2 mg/kg). We found that 143B tumors administered combination-treated formed substantially smaller tumors in nude mice compared with the vehicle and either drug alone. Combination-treated formed substantially smaller tumor (Figure 8A and 8B). Both cinobufagin and CDDP significantly inhibited tumor growth compared with vehicle, and combined cinobufagin + CDDP treatment led to a significantly larger inhibitory effect than either compound alone (Figure 8A and 8B). Furthermore, combination administration improved mice survival at week ten (Figure 8C). Combined cinobufagin and CDDP administration did not significantly affect mice body weight compared with without tumor burdens, indicating the relative safety of this regimen (Figure 8D). In addition, tumor metastasis were found in the lungs according to the HE staining (Figure 8E), and we found that combined cinobufagin and CDDP group compared with the vehicle and agent alone, regardless of the number or volume with metastases were significantly less and smaller (Figure 8F and 8G). In addition, apoptosis related mRNA and proteins were detected from xenograft mice of tumor sections. We tested the expressions of Bax, Bcl-2, Caspase-3 and Caspase-9 respectively. RT-PCR analysis showed alterations in the levels of expression of Bax (pro-apoptotic) and Bcl-2 (anti-apoptotic), resulting in a significant increase in Bax after treatment of the 143B xenografts with CDDP plus cinobufagin, and the Bcl-2 shown opposite result (Figure 8H). The relative mRNA expression of Caspase-3 and Caspase-9 of mice tumor tissues was highest in cinobufagin + CDDP group followed by CDDP alone, cinobufagin alone, and vehicle in that order (Figure 8H). Subsequently, Immunoblotting experiments were performed and the results were similar to RT-PCR as shown in Figure 8I. Hence, this combination regimen may provide a relatively safe and effective therapeutic option for the treatment of OS as demonstrated in the animal model.

Then we determined if therapeutic alliance reduced Notch pathway activity *in vivo* by analyses of Notch1 and its target genes, Hes1, Hes5, and Hey-L in xenograft mice (Figure 8J-8L). Consistent with the *in vitro* data, there were reduction of NICD1, Hes1, Hes5 and Hey-L mRNA levels and NICD1 protein after combined treatment compared with the vehicle.

Thus, all these results indicated that CDDP in combination with cinobufagin treatment could markedly inhibit 143B xenograft growth *in vivo* and improve nude mice survival.

## DISCUSSION

The development of cancer involves a complex interplay among cellular processes, a variety of cancer promoting factors are involved. Researchers have tried many ways to improve efficacy of the chemotherapeutic agents, such as modifying the chemical structure of drugs or altering the mode of administration, etc. Though all these attempts, treatment with a single drug is rarely effective. Combination therapy is now considered to be a standard approach to chemotherapy [10, 20, 22, 27-29]. Numbers of advantages of combination therapy have been found, which include: I) targeting of two or more critical molecular processes and/or pathways; II) Reducing the chemoresistance of tumor cells; III) delivery of lower dose agents associated with lower toxicity; IV) increasing patients’ tolerance. The superiority and effectiveness of combination chemotherapy has stimulated interest in exploring drugs with different modes of activity at lower dosages [10, 20, 27]. Cinobufagin, as a Chinese medicine, has been widely investigated both *in vitro* and *in vivo* [23-35, 30-32]. Recently, a lot of studies showed that cinobufagin could induce apoptosis of cancer cells, including blood lymphocytes, hepatocellular carcinoma, colorectal cancer, non-small cell lung cancer, breast cancer and ovarian cancer, and may be an effective therapeutic agent that can reduce toxicity and side effects *in vivo* [23, 25, 26, 31-38]. Cinobufagin has been shown to induce reversal of P-gp-mediated multidrug resistance in colon cancer and has been developed into a safe and potent P-gp modulator for combination use with anticancer drugs in cancer chemotherapy [39]. Zhang *et al.* [25] demonstrated that cinobufagin inhibits tumor growth by inducing intrinsic apoptosis through the AKT signaling pathway in human non-small cell lung cancer cells. However, little is known about its effects on OS cells.

CDDP has a broad range of antitumor activity in malignant disease and widely used to treat many types of cancer. Considering its toxicity and side effects, the clinical use of CDDP is limited by dose for safety profile, several strategies have been consider enhancing the clinical activity of CDDP, especially for use in combination therapy [20, 29, 40].

A previous study reported that the results of a study on the combination of Oldenlandia diffusa (traditional Chinese medicine) and CDDP indicated that the combination of these two drugs had a synergistic effect in inducing apoptosis and reduced the dose of CDDP required, as well as its toxic side effects [11]. Inspired by this, we have performed the experiment of applying cinobufagin to OS cells. Cinobufagin, a natural, active compound isolated from giant toads (Chan Su) [41], is known to induce apoptosis in several cancer cell types by activating both the extrinsic and intrinsic pathways of apoptosis in tumors [25, 41, 42]. However, little is known about the effects of cinobufagin on OS cells. In this study, we tested the combined effects of cinobufagin on CDDP-treated OS 143B cells using CCK-8 and FACS assays. We found that cinobufagin inhibits the growth and proliferation of CDDP-treated 143B cells in a dose- and time-dependent characteristics. Furthermore, cinobufagin plus CDDP was found to increase the therapeutic efficacy of each drug, which exhibiting a broad range of synergistic interaction (CI<1) in 143B cells. Most importantly, the combination of cinobufagin and CDDP resulted in appreciable DRIs ranging from 2.9- to 7.2-fold dose reductions. Since a lower concerntration of two drugs can produce a given effect with reduced toxicity and side effects in normal tissues, this surprising result hints that cinobufagin plus CDDP has the potential to be developed as a therapeutic schedule for OS patients and reduce the adverse reaction. In addition, cinobufagin enhanced the CDDP-induced apoptosis of 143B cells, as evidenced by Annexin V-FITC/7-AAD and Hoechst 33258 staining assays, and by western blotting. Compared to cinobufagin or CDDP alone, low doses of these two drugs in combination induced substantial apoptosis of 143B cells. Similarly, cinobufagin plus relatively low dose of CDDP could enhance arrest of tumors in S phase of the cell cycle, and expression of cell cycle-related proteins also confirmed this result. This is also one of the mechanisms leading to inhibition of cell proliferation. Furthermore, *in vivo* experiments, we found that combined treatment with these two drugs effectively reduced tumor growth in 143B xenograft mice without affecting their weight, which shows that this combination is relatively safe and has less systemic toxicity in the animals. Based on the synergistic antitumor activity profiles of combined cinobufagin and CDDP treatments both *in vitro* and *in vivo* and the absence of cytotoxicity, we believe that CDDP has greater therapeutic value when used in with cinobufagin against OS.

It is well known that cancer invasion and metastasis is a complicated multistep process involving numerous effector molecules. The degradation of extracellular matrix is an essential step in cancer invasion and metastasis [43]. A large number of literature studies shown that VEGF, MMP-2 and MMP-9 have been regarded as metastasis-related genes, which play important roles in cancer invasion and metastasis [43-48]. In present study, we examined the expression of related mRNA and proteins and found that cinobufagin and CDDP inhibited both the expression and activities of MMP-2 and MMP-9 as well as VEGF in 143B OS cells *in vitro*. Studies have shown that extracellular matrix (ECM) degradation and neovascularization are the basis characteristic of tumor growth, invasion and metastasis [45, 46]. MMP-2 and MMP-9 could degrade the ECM and basement membrane collagen of blood vessels then promote tumor cell invasion and matastasis, while vascular endothelial growth by binding to VEGF receptor to promote angiogenesis, thus participating in the development and progression of tumors. Indeed, our *in vitro* results showed that combination cinobufagin and CDDP inhibited migration and invasion of OS cells through the Matrigel™. The animal model employed in our study was a close mimic of lung metastasis of human OS. This model was established using the 143B OS cell line, which has a high tendency to metastasize to the lung and is used by many scholars working in the same field [49-51]. In the present study, in which the primary tumor was resected at the end of the experiment, all mice in the vehicle group exhibited 100% lung metastasis, confirming the model to be ideal for studying tumor with lung metastasis. In contrast, in the combined treatment group we observed not only the smallest volume but also the least amount of lung metastases; this result is consistent with the results of our *in vitro* study. It might account for the underlying mechanism of the synergistic effect repression of primary OS and the reduced metastatic potential observed following treatment by the combination of cinobufagin and CDDP. Although these results are inspiring, the antitumor effects of this combination should be investigated in more OS cell lines and in patients in future clinical studies.

**Figure 9:**
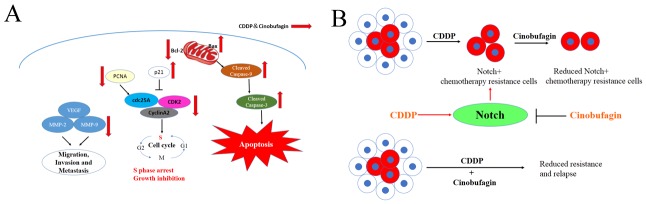
Schematic view depicting mechanisms of action of combination cinobufagin and CDDP **(A)** Signaling pathways for anti-tumor effects by cinobufagin and CDDP. The combination of cinobufagin and CDDP induced the cellular apoptosis and S phase arrest through Bax/Bcl-2 and p21, Cyclin A2/CDK2/cdc25A pathways. Meanwhile, the combination decreased VEGF, MMP-2 and MMP-9 to inhibit cellular migration, invasion and metastasis. **(B)** Diagram illustrating that CDDP does not target Notch+ chemotherapy resistance cells, but cinobufagin could partially eliminated Notch+ chemotherapy resistance cells, so as to play a synergistic anticancer effect with CDDP and emphase the need to target residual drug-resistant cells to eliminate all cancer cells.

Although we are not the first to demonstrate that cinobufagin could inhibit the survival of human 143B OS cells, our findings are more specific in demonstrating that it acts by enhancing preexisting CDDP-induced apoptotic processes, cell cycle arrest, and decreased cell motility (Figure 9A). In addition, we have also demonstrated, indeed for the first time, that combination cinobufagin and CDDP inhibited OS *in vitro* and *in vivo*, which was associated with suppression of Notch signaling pathways (Figure 9B), which consistent with our previous study [24]. These pathways have been shown to play major roles in OS carcinogenesis [52]. As the Notch signaling pathway plays a key role in cell proliferation, survival, apoptosis and differentiation, the Notch signaling pathway could be a promising target for tumor target gene therapy [52, 53]. Cinobufagin as a regulatory factor for Notch signaling pathway, is less toxic, has fewer side effects and is more economically accessible than other regulatory factors such as GSI (γ-secretase inhibitor) and inhibitors. The combination of cinobufagin and CDDP in OS cells can enhance the lethality of cancer cells, which may be due to downregulation of Notch signaling in the resistance of cancer cells. Cinobufagin inhibits the expression of Notch signaling, so as to eliminate the resistance of cancer cells to CDDP, and thus exert a synergistic anticancer effect in combination with CDDP. The combination of cinobufagin and CDDP is able to overcome the chemotherapy resistance caused by Notch overexpression, so this combination therapy not only improves chemosensitivity but also reduces the toxicity and side effects of chemotherapy because a lower dose is required.

In summary, our present study has demonstrated that cinobufagin in combination with CDDP was able to markedly inhibit cell activation, migration and invasion, and increase apoptosis and cell cycle arrest in the S phase *in vitro*, as well as suppressing xenograft tumor growth and metastasis and improving survival rate *in vivo*, compared to cinobufagin or CDDP treatment alone, demonstrating a synergistic effect. Our results also showed that its mechanism was related to downregulation of Notch signaling. Taken together, our findings suggest that cinobufagin plus CDDP is a promising candidate regime for the treatment of human OS.

## CONCLUSIONS

In conclusion, our study provides important information regarding the antitumor activities of cinobufagin and CDDP in human OS. Specifically, we have demonstrated that combination cinobufagin and CDDP are useful in potentiating antitumor activity *in vitro* and *in vivo* by suppressing Notch signaling pathways and thus suppressing OS growth. Cinobufagin as a traditional Chinese medicine might represent an adjuvant second-line antitumor drug, which might enhance CDDP cytotoxicity, and then CDDP remains as the first-line antitumor medication for chemotherapeutic agent in clinical treatment. Consequently, we hypothesize that cinobufagin plus CDDP could be a promising approach for the treatment of human OS.

## MATERIALS AND METHODS

### Ethics statement

The study protocol was approved by the Experimental Animal Care Committee of Renmin Hospital of Wuhan University and conforms with the provisions of the Declaration of Helsinki.

### Chemicals and reagents

Cinobufagin and CDDP were purchased from from Sigma (St. Louis, MO, USA). All media were obtained from GE Healthcare (Hyclone, UT, USA). Fetal bovine serum was from Gibco (Australia). Cell Counting Kit-8 (CCK-8) was obtained from Dojindo (Kumamoto, Japan). Primary antibody for Notch-1 intracellular domain (NICD1), Hes1, VEGF, Bcl-2 and Bax were from Abcam (Cambridge, UK). RIPA Lysis Buffer, primary antibodies against, Cleaved Caspase-9, Cleaved Caspase-3, MMP-2, MMP-9 and horseradish peroxidase (HRP)-labeled secondary antibody were from Cell Signaling Technology (Beverly, MA, USA). Amersham ECL Western blotting detection reagents and analysis system was purchased from GE Healthcare (Hyclone, UT, USA) and all other chemicals were from Sigma (St. Louis, MO, USA), unless otherwise indicated.

### Cell culture

The human OS cell line 143B were obtained from the China Center for Type Culture Collection (Wuhan, China). The cells were grown in α-MEM supplemented with 10% fetal bovine serum (FBS) and 1% antibiotics mixture (streptomycin 100 μg/ml, penicillin 100 U/ml). Cells were incubation in a humidified environment at 37°C containing 5% CO_2_. Culture medium was replaced every three days.

### Cell viability and cytotoxicity assay

Briefly, 143B (5 × 10^3^ cells/well) cells were suspended and cultured in quadruple in a 96-well plate. The cells viability was detected using the CCK-8 assay at indicated time points. Then, CCK-8 (10 μl) was added to each well containing 100 μl mixture of culture medium and further incubated for 1.5 h in dark at 37°C. Cells viability was measured by auto microplate reader (Tecan Sunrise, Austria) when using 450 nm absorbance. Cell viability was calculated according to the protocol.

### DAPI staining assay

Cell proliferation can be tested by DAPI (4’, 6-diamidino-2-phenylindole) staining assay. 143B (1×10^5^ cells/well) cells were inoculated in 6-well plate which containing 10 mm coverglass and subsequent treatment for cinobufagin and CDDP alone and in combination for 48 h. Then cells were fixed with 4% paraformaldehyde for 30 min and washed in phosphate-buffered saline (PBS), stained with DAPI and washed for the second with PBS. Finally, the images were photographed by an inverted fluorescence microscope (Olympus).

### Flow cytometry

For cell cycle analysis, briefly, cells were harvested and then fixed with 70% (V/V) ethanol, treated with 300 μg/ml RNase A, and 10 μg/ml propidium iodide were added. After incubation for 30 min at normal temperature in dark, the cells were examined by FCM (Bectom-Dickinson, San Jose, CA, USA). Data were analysed using Modfit software. The apoptosis assay was performed with an Annexin V-FITC (fluorescein isothiocyanate, FITC) / 7-AAD (7-Aminoactinomycin D) apoptosis detection kit (Sigma-Aldrich) according to the protocol. Cells were harvested resuspended in 1 × binding buffer with a concentration of 1 × 10^6^ cells/ml. Then, Annexin V-FITC conjugate (5 μl) and 7-AAD solution (5 μl) were added to each tube containing 500 μl mixture of 1 × binding buffer. Cells were stained for 15 min at 25°C away from light. Stained samples were analyzed using FCM and the apoptosis rate was determined using Flowjo software.

### Hoechst 33258 for apoptosis

143B cells were seeded in 6-well plates and treated with cinobufagin and CDDP alone and in combination for 48 h. Then, each well were stained according to the protocol of Hoechst 33258 Staining Kit (Sigma). After incubation for 30 minutes away from light, the cells were photographed with an inverted fluorescence microscope. bright-blue fluorescent and condensed nuclei were designated as apoptotic cells.

### Scratch wound-healing motility assay

143B cells were seeded in 6-well plates and if the cells were confluence, wound-healing assay can perform with a 1000-μL sterile micropipette tip to scratch the cell monolayer. Then, the cells were treatment with different conditions for 24 and 48 h with serum free medium respectively, the plates were washed thrice with PBS and add fresh serum-free medium at indicated time. Images of the scratch area with or without treatment were photographed and measured at indicated times under an inverted microscope. Five randomly selected fields in the scratch area. The cell migration capability was calculated based on the percentage (indicated time’s scratch width/original scratch width).

### *In vitro* cell invasion and migration assays

Transwell membranes (Corning Inc., New York, NY, USA) were used in the cell invasion assays. Briefly, 143B cells were treated with cinobufagin and CDDP alone and in combination for 48 h. After treatment, 2×10^5^ cells in 250 μL were seeded into the upper chambers pro-coated with Matrigel™ (2.5mg/ml) without FBS medium in triplicate. The medium with 10% FBS was added to the lower chamber served as a chemo-attractant. And then incubated for 48 h, cells on the upper surface were wiped with a cotton swab, cells migrating to the lower surface of the membrane were fixed with 4% paraformaldehyde for 20min, and stained with 0.1% crystal violet for 10 min, and then washed with PBS for twice. Five randomly selected fields by microscopy (×200). photomicrographs were taken and the number of cells were calculated. All experiments were repeated thrice.

Cell migration assays were performed for the second time using Transwell membranes. The empirical procedure was more or less likely to the assay of cell invasion, except for the upper chamber were not pro-coated with Matrigel™.

### Enzyme-linked immunosorbent assay

Cells were seeded in Petri dish, and treated with cinobufagin and CDDP alone or in combination when the cells were in logarithmic phase of growth for 48 h, the supernatant of culture medium were harvested and centrifuged at ×3000g for 15min at room temperature to remove cell debris and stored at -80°C until the ELISA (Abcam, Cambridge, UK) was performed. The detail procedures were undertook according to the manufacturer’s protocol.

### Gelatin zymography analysis of MMPs activity

Cells were treated with cinobufagin and CDDP alone or in combination for 48 h in serum-free medium. the supernatant of culture medium were harvested and centrifuged to remove cell debris. The activities of MMP-2 and MMP-9 were tested by gelatin zymography briefly according to the protocol as follow. Samples were applied to sodium dodecyl sulfate (SDS) polyacrylamide gel electrophoresis (PAGE) containing a 10% gel and 0.1% gelatin. After electrophoresis, the gel was washed in 2.5% Triton X-100 to remove the SDS. And then incubated in developing buffer at 37°C for 24 h and stained with Coomassie blue (0.05%), enzymatic activities were visualized as clear bands in the blue background.

### Reverse transcription quantitative real-time PCR

Quantitative real-time PCR (RT-qRCR) was done according standard techniques, as described previously [4], The gene specific primers used are listed in Table 4.

**Table 4:** Primer sequences used for real-time PCR analysis

Genes	Primer sequence	
Ki67	Forward	5’- GGCACTTTCTGTGAGGAGGAC-3’
	Reverse	5’-GCAGTCAGGCGTGTTGTTCT-3’
PCNA	Forward	5’- ATTCTGGAAATGACAGTGAAGCAC-3’
	Reverse	5’-CACCTCGGTATTAACGCCCTC-3’
Bax	Forward	5’-GAAGCCGGTGGTGGAGAA-3’
	Reverse	5’-GCTTGGAGTTGGGCTGGTG-3’
Bcl-2	Forward	5’- GAAGCAGGTAATGGAGCAAGGA-3’
	Reverse	5’-GAAGCGTAGTTGTTGAGATGCG-3’
Caspase-3	Forward	5’-GCGGTTGTAGAAGTTAATAAAGGTA-3’
	Reverse	5’-CATGGCACAAAGCGACTGG-3’
Caspase-9	Forward	5’-TGCTCAGACCAGAGATTCGC-3’
	Reverse	5’-TCTTTCTGCTCGACATCACCAA-3’
VEGF	Forward	5’-ACTTTGGTATCGTGGAAGGACTCAT-3’
	Reverse	5’-GTTTTTCTAGACGGCAGGTCAGG-3’
MMP-2	Forward	5’-GAGTGCATGAACCAACCAGC-3’
	Reverse	5’-AAACTTGCAGGGCTGTCCTT-3’
MMP-9	Forward	5’-TCTATGGTCCTCGCCCTGAA-3’
	Reverse	5’-TTGTATCCGGCAAACTGGCT-3’
Notch1	Forward	5’-GGCACTTTCTGTGAGGAGGAC-3’
	Reverse	5’-GCAGTCAGGCGTGTTGTTCT-3’
Hes1	Forward	5’-CAGATCAATGCCATGACCTACC-3’
	Reverse	5’-AGCCTCCAAACACCTTAGCC-3’
Hes5	Forward	5’-AGCCCCAAAGAGAAAAACCGACTG-3’
	Reverse	5’-TGGAGCGTCAGGAACTGCACGG-3’
Hey-L	Forward	5’-ACCGCATCAACAGTAGCCTTTCT-3’
	Reverse	5’-GCATTTTCAAGTGATCCACCGTC-3’
GAPDH	Forward	5’-ACTTTGGTATCGTGGAAGGACTCAT-3’
	Reverse	5’-GTTTTTCTAGACGGCAGGTCAGG-3’

### Western-blot analysis

For Western blot analysis, cells were harvested and lysed in 1×RIPA buffer containing protease inhibitor cocktails (Roche). The detail protocol for Western blotting as described previously [4]. Primary antibodies were incubated overnight at 4°C. Primary antibodies used were anti-Ki67 (1:1000), anti-PCNA (1:1000), anti-NICD1 (1:500), anti-Hes1 (1:500), anti-Cleaved Caspase-9(1:1000), anti-Cleaved Caspase-3 (1:1000), and anti-β-Actin or anti-GAPDH (1:2000). Secondary antibodies (1:3000) conjugated with horseradish peroxidasewere were incubated for 1.5h at room temperature. Subsequently, analyzed by enhance chemiluminescence substrate.

### Caspase-3 and Caspase-9 activity assay

Caspase-3 and Caspase-9 activity assay was performed by Caspase-Glo® 3, 9 Assay kit (Sigma) in 96-well plates. Lysates of 143B cells were prepared after treated with cinobufagin and CDDP alone and in combination for 48 h. Assessment of caspase-3 and Caspase-9 activity was followed as instruction. All experiments were repeated thrice.

### 143B tumor xenograft model in nude mice

Male BALB/c-nu/nu nude mice were obtained from Center for Animal Experiment of Wuhan University and housed in laminar flow cabinets under specific pathogen-free conditions with food and water *ad libitum*. Xenograft models in nude mice were done according standard operating procedures, as described in our previously study [24]. The animals were randomly divided into 5 groups with 6 mice each group: (a) Health mice. Without tumor burden. (b) vehicle; (c) Cinobufagin. Cinobufagin (5 mg/kg) was injected intraperitoneally (i.p.) every other day for 3 weeks; (d) CDDP. CDDP (4 mg/kg) was administered i.p. twice a week for 3 weeks. (e) Combined treatment group. 3 mg/kg of cinobufagin and 2 mg/kg CDDP respectively was administered according to aforementioned. The tumor volumes (V) were determined once a week until to 8 weeks by caliper measurement and used the formula: V = length × (width)^2^/2. The weight (g), survival (at week 10) and toxicity relevant to treatment were also recorded and calculated. Xenotransplanted tumors and lungs were harvested for additional analysis as described below.

### HE staining

The lung tissue were separated and fixed with 4% paraformaldehyde. After 24h, paraffin embedded, 4 μm discontinuous slice, hematoxylin and eosin (HE) staining, morphological changes of lung tissue sections were observed under microscope.

### Immunofluorescence assay

Briefly, the cells were fixed with 4% paraformaldehyde for 30min, immunofluorescence was assessed using anti-Hes1 antibody (1:400 dilution) incubated at 4°C overnight. After that secondary antibody was added for 2 h at room temperature. Each step of the procedure was followed by a PBS wash, and the DAPI were counterstained. Finally, we observed and photographed under fluorescence microscope.

### Statistical analyses

SPSS 13.0 statistical software package was used to perform all statistical analysis. All experimental values are expressed as the mean ± standard deviation (SD) of at least three independent experiments. Statistical significant different between samples using the Student’s t-test. P<0.05 was considered as statistically significant.
